# Distinct frontal lobe morphology in girls and boys with ADHD

**DOI:** 10.1016/j.nicl.2014.12.010

**Published:** 2014-12-10

**Authors:** Benjamin Dirlikov, Keri Shiels Rosch, Deana Crocetti, Martha B. Denckla, E. Mark Mahone, Stewart H. Mostofsky

**Affiliations:** a Center for Neurodevelopmental and Imaging Research, Kennedy Krieger Institute, Baltimore, MD, USA; b Department of Neuropsychology, Kennedy Krieger Institute, Baltimore, MD, USA; c Department of Neurology, Johns Hopkins University School of Medicine, Baltimore, MD, USA; d Department of Psychiatry and Behavioral Sciences, Johns Hopkins University School of Medicine, Baltimore, USA; e Department of Pediatrics, Johns Hopkins University School of Medicine, Baltimore, MD, USA

**Keywords:** ADHD sex-differences, Cortical morphology, Development, Frontal lobe

## Abstract

**Objective:**

This study investigated whether frontal lobe cortical morphology differs for boys and girls with ADHD (ages 8–12 years) in comparison to typically developing (TD) peers.

**Method:**

Participants included 226 children between the ages of 8–12 including 93 children with ADHD (29 girls) and 133 TD children (42 girls) for which 3T MPRAGE MRI scans were obtained. A fully automated frontal lobe atlas was used to generate functionally distinct frontal subdivisions, with surface area (SA) and cortical thickness (CT) assessed in each region. Analyses focused on overall diagnostic differences as well as examinations of the effect of diagnosis within boys and girls.

**Results:**

Girls, but not boys, with ADHD showed overall reductions in total prefrontal cortex (PFC) SA. Localization revealed that girls showed widely distributed reductions in the bilateral dorsolateral PFC, left inferior lateral PFC, right medial PFC, right orbitofrontal cortex, and left anterior cingulate; and boys showed reduced SA only in the right anterior cingulate and left medial PFC. In contrast, boys, but not girls, with ADHD showed overall reductions in total premotor cortex (PMC) SA. Further localization revealed that in boys, premotor reductions were observed in bilateral lateral PMC regions; and in girls reductions were observed in bilateral supplementary motor complex. In line with diagnostic group differences, PMC and PFC SAs were inversely correlated with symptom severity in both girls and boys with ADHD.

**Conclusions:**

These results elucidate sex-based differences in cortical morphology of functional subdivisions of the frontal lobe and provide additional evidence of associations among SA and symptom severity in children with ADHD.

## Highlights

• Children with ADHD showed reductions in cortical surface area.
• Boys with ADHD showed overall reductions in total premotor cortex surface area.
• Girls with ADHD showed overall reductions in total prefrontal cortex surface area.
• Surface area in the ADHD group was associated with symptom severity.

## 1 Introduction

Attention-Deficit/Hyperactivity Disorder (ADHD) is among the most common childhood disorders, characterized by developmentally inappropriate levels of hyperactivity, impulsivity, and inattention. Research on children with ADHD primarily focuses on boys due to the disproportionate prevalence of boys over girls with ADHD (2:1 to 9:1) (Rucklidge, 2010), however the rate of diagnosis among girls is rapidly increasing (Mahone, 2012). A growing body of research has focused specifically on comparing girls with ADHD to typically developing (TD) girls (Hinshaw et al., 2007; Wodka et al., 2008), but few studies have examined sex differences in large samples of boys and girls with ADHD in comparison to each other and same-sex peers. In particular, sex differences in brain structure and function in children with ADHD have rarely been studied (cf. Castellanos et al., 2002; Mahone et al., 2011) whereas there has been more research on behavioral functioning in boys and girls with ADHD.

The limited literature on sex differences in ADHD has shown that boys and girls with ADHD differ in terms of clinical presentation and, to some extent, neuropsychological functioning. Clinically, boys are more commonly diagnosed with the combined subtype and show a greater preponderance of hyperactive/impulsive symptoms, whereas girls with ADHD are more often diagnosed with the inattentive subtype (Hinshaw et al., 2006). In addition, there is some evidence that boys and girls diagnosed with ADHD in childhood tend to have different functional outcomes (Rucklidge, 2010). Research examining sex differences in neuropsychological functioning suggests that, compared to age and sex-matched controls, both boys and girls with ADHD show impairments in executive function (O'Brien et al., 2010), greater multitask interference (Ewen et al., 2012), and weaker skeletomotor control (Mostofsky et al., 2003), cognitive control (Mostofsky et al., 2003; Rubia et al., 2001), and emotional control (Castellanos et al., 2006). Boys with ADHD tend to display greater motor impairment, including persistence of motor subtle signs (increased dysrhythmia) into late childhood (Cole et al., 2008; MacNeil et al., 2011), greater impairment during effortful response inhibition (O'Brien et al., 2010), and slower execution of timed movements (Denckla and Rudel, 1978). There is also some evidence that girls with ADHD tend to show greater higher order cognitive deficits during childhood, such as impairments in planning (O'Brien et al., 2010). In sum, sex differences in clinical presentation may be related to greater motor impairments in boys with ADHD and different executive function profiles for girls and boys with ADHD.

Research examining neuroanatomical differences in children with ADHD is also predominated by male samples, limiting the opportunity to examine sex differences. In general, these studies have shown regional abnormalities in the frontal cortex (Arnsten, 2009; Seidman et al., 2005; Sowell et al., 2003), prefrontal and premotor cortex (Mostofsky et al., 2002), supplementary motor cortex (Mahone et al., 2011), cerebellum (Berquin et al., 1998; Mostofsky et al., 1998), and basal ganglia (Qiu et al., 2009) as well as parietal and temporal cortices (Shaw et al., 2006; Wolosin et al., 2009). Longitudinal studies report that, although the cortical development in ADHD follows a pattern similar to that of TD children, in which primary sensory and motor regions develop before higher-order association areas, peak cortical thickness (CT) occurs before peak cortical surface area (SA) and girls develop earlier than boys, children with ADHD show a delay of several years in both peak CT and SA that normalizes by age 15–17 (Shaw et al., 2012, 2007). Furthermore, the rate at which children with ADHD normalize to TD CT levels is associated with outcome (Shaw et al., 2006). Additionally, children with ADHD show a disruption of typically developing asymmetries, particularly in the orbito-inferior frontal gyral region (Shaw et al., 2009). Cortical asymmetries in typically developing adults show sensitivity to sex differences with a leftward bias, particularly premotor, in males and mixed results in females (Goldberg et al., 2013; Koelkebeck et al., 2014; Shaw et al., 2009). Despite these observations, very little work has been done investigating sex-based differences in cortical asymmetry in ADHD, while many studies of cortical morphology in ADHD have included girls in their analyses, few studies have examined or reported sex differences in ADHD, possibly due to insufficient sample size and a lack of statistical power (e.g., Almeida Montes et al., 2013).

Very few studies have examined sex differences in neuroanatomy among children with ADHD. Studies that have compared girls with ADHD to TD girls found evidence of smaller total brain volume and cerebellar volume in girls with ADHD (ages 5–16 years) (Castellanos et al., 2001) and reduced gray matter density in the right cerebellum in girls with ADHD (age 8–10 years) (Montes et al., 2011). Only two studies have compared relatively large samples of girls and boys with ADHD to same-sex TD children. Castellanos et al. (2002) applied a whole brain approach in children ages 5–18 years and did not find evidence of ADHD-related sex differences in gray and white matter in the frontal, temporal, parietal and occipital lobes or the basal ganglia or cerebellum; however, the authors note the need for approaches to detect more localized abnormalities in the frontal lobe. Mahone et al. (2011) performed a detailed examination of frontal lobe morphology in children ages 8–12 years, reporting reduced gray matter volume in the left lateral premotor cortex in girls with ADHD while boys with ADHD showed reduced white matter volume in the left medial PFC.

The current study builds upon the limited research on sex differences in cortical morphology in children with ADHD by directly comparing large samples of boys and girls with ADHD, matched on ADHD subtype, to same-sex TD peers. Furthermore, we examined functionally distinct sub-regions of the frontal lobe using a newly developed automated atlas (Ranta et al., 2014). The use of this atlas facilitates grouping regions of interest (ROIs) by function (i.e., premotor and prefrontal) and investigating the effects of diagnosis and sex for each functional subdivision. We hypothesized that: 1) children with ADHD would show regional reductions in cortical morphology compared to age matched TD children, 2) boys with ADHD would show greater premotor differences compared to TD boys, and 3) cortical morphology would be associated with symptom severity within the ADHD group.

## 2 Method

### 2.1 Participants

Participants included 226 children, ages 8–12 years. The ADHD group comprised 93 children (29 girls), representing all three subtypes. The remaining 133 subjects (42 girls) comprised the typically developing (TD) group. The demographic information is summarized in Table 1.

Participants were recruited so as to provide a representative community sample. Recruitment was principally through advertisement in local public and private schools, with additional recruitment through community-wide advertisement, volunteer organizations, medical institutions, and word of mouth. A brief telephone interview was conducted with a parent to determine whether their child met the initial inclusion criteria. Next, a structured diagnostic interview using the Diagnostic Interview for Children and Adolescents-IV (DICA-IV; Reich et al., 1997) was conducted over the phone with the child's parent. Participants were then scheduled for a study visit and were mailed the Conners' Parent and Teacher Rating Scales-Revised Long Version (CPRS-R:L; Conners et al., 1998), and the ADHD Rating Scale-IV, home and school versions (ADHD-RS; DuPaul et al., 1998) to confirm diagnostic status. During the initial study visit, participants were administered the Wechsler Intelligence Scale for Children-IV (Wechsler, 2003) and the Word Reading subtest from the WIAT-II (Wechsler, 2002) in order to rule out intellectual and reading disabilities.

Exclusion criteria for the all participants consisted of the following: (1) Full Scale IQ (FSIQ) score below 80 based on the WISC-IV, (2) history of intellectual disability, seizures, traumatic brain injury or other neurological illnesses, (3) psychotropic medications (other than stimulant medication for children with ADHD), (4) a WIAT-II Basic Reading score below 85, and (5) a DICA-IV diagnosis of Conduct Disorder, Mood Disorder, Generalized Anxiety Disorder, Separation Anxiety Disorder or Obsessive Compulsive Disorders. Given the high rates of comorbidity between ADHD and Oppositional Defiant Disorder (ODD) (Jensen et al., 1999) and because we did not have specific predictions about the impact of comorbid ODD in children with ADHD, children with comorbid ODD were not excluded. We also did not exclude TD or ADHD participants based on the presence of a Specific Phobia.

An ADHD diagnosis was based on the following criteria: (1) an ADHD diagnosis on the DICA-IV psychiatric interview and (2) a T-score of 65 or higher on the DSM-IV:inattentive or DSM-IV:hyperactive–impulsive scales on the CPRS-R:L or a score of 2 or 3 on at least 6 out of 9 items on the Inattentive or Hyperactivity/Impulsivity scales of the ADHD-RS. This information was then reviewed by a child neurologist along with consideration of teacher ratings for a final confirmation. Participants in the TD group could not meet diagnostic criteria for any psychiatric disorder, other than Specific Phobia, based on DICA-IV and their scores had to be below clinical cutoff scores on the parent-report measures (CPRS-R:L and ADHD-RS). Participants with ADHD taking stimulant medication were asked to withhold medication on the day prior and day of testing.

This study was approved by the Hospital Medical Institutional Review Board. Written consent was obtained from a parent/guardian and assent was obtained from the participating child.

### 2.2 MRI acquisition and processing

Before each scanning session the participants completed a practice scanning session to acquaint themselves with the scanning environment. Participants entered the mock scanner room with an instructor and were guided through the sequence of events that occur on the day of their actual scan, including sliding into the scanner, wearing ear plugs, hearing loud MRI scanner noises, and being alone in the scanner for 10 min.

All scanning acquisition was completed using a 3.0 T Philips 3T ‘Achieva’ MRI scanner (Best, The Netherlands). MPRAGE images (Slice thickness = 1.0 mm; FOV = 26 cm; Matrix size: 256 × 256) were checked for motion and only images with minimal motion were used for FreeSurfer processing. Atlas based regions of interest (ROIs) and total cerebral volume measurements were obtained using FreeSurfer (Fischl et al., 2004). Within FreeSurfer, ROIs were delineated using a novel automated frontal lobe atlas, the Ranta atlas (Ranta et al., 2014). Compared to the Desikan atlas, the Ranta atlas is based on functionally distinct regions of interest that were manually delineated using a pediatric population (8–12 years old). The Ranta frontal lobe atlas includes the left and right hemisphere anterior cingulate (ACC), dorsal lateral prefrontal cortex (DLPFC), medial prefrontal cortex (mPFC), inferior lateral prefrontal cortex (ILPFC), medial orbitofrontal cortex, lateral orbitofrontal cortex, frontal eye field (FEF), lateral premotor cortex (LPM), supplementary motor complex (SMC), and primary motor cortex (M1). The lateral and medial OFC were combined to create a single orbitofrontal cortex (OFC) ROI. Ranta ROIs were combined to permit examination of prefrontal cortex (PFC) ROIs (ACC, DLPFC, mPFC, ILPFC, and OFC) and premotor cortex (PMC) ROIs (FEF, LPM, and SMC) collectively. SA and CT for each ROI were extracted using FreeSurfer. FreeSurfer parcellation quality was visually inspected for each subject.

### 2.3 Data analysis

Data analysis was accomplished using SPSS Statistical Version 20 (IBM, Chicago). In line with previous research, each participant's frontal lobe ROI cortical measurements were normalized by multiplying the raw cortical metric by the ratio of their respective diagnostic group's average total brain volume (TBV) and individual subject's total brain volume (e.g., [Subject ROI SA ∗ Mean ADHD TBV]/Subject TBV) (Kramer et al., 2007; Mahone et al., 2011; Ranta et al., 2009). Normalization was done to account for common findings of reduced cerebral/frontal gray matter volume in children with ADHD. More stringent methods of account for TBV (e.g., covarying for TBV) were not employed due to the expectation of subtle group differences. Separate 2 Diagnosis × 2 Sex × 2 Hemisphere × 3 (PMC) or 5 (PFC) ROI repeated measure ANOVAs were run for CT and SA, resulting in four total models. Hemisphere was included in the model due to evidence of abnormal cortical asymmetries in ADHD as well as anatomical differences in cortical lateralization in boys and girls that may underlie functional differences seen in ADHD (Goldberg et al., 2013; Koelkebeck et al., 2014; Shaw et al., 2009). The normalized scores for PFC and PMC ROIs were used as dependent variables in their respective models. Only effects involving diagnosis as a main effect or in interaction were reported and interpreted. Univariate ANOVAs were used to further investigate significant main effects and interactions. Additionally, due to the high prevalence of comorbid ODD and participants prescribed stimulant medication at the time of the study among our sample of children with ADHD, we repeated our analysis for children currently prescribed stimulant medication and those not currently prescribed stimulant medication, as well as those children with and without comorbid ODD to test the effect of both medication and ODD status.

A hierarchical approach was used to assess the relationship between symptom severity, as measured by the CPRS-R:L ADHD Total Score, and SA in the ADHD group only. Since correlations with symptom severity were only tested in the ADHD group, unnormalized scores of SA were used. First, partial correlations were used to investigate associations between CPRS-R:L raw total score and PMC and PFC morphology while controlling for age. Second, for significant associations, follow-up partial correlations were used to investigate the association between CPRS-R:L raw total score and PFC and PMC ROIs within sex.

## 3 Results

### 3.1 Sample characteristics

There were no significant diagnostic differences within sex or at the whole group level (ADHD vs. TD) for age, socio-economic status, sex, WISC-IV Perceptual Reasoning Index (PRI), and Edinburgh Handedness Inventory. Representation of the ADHD subtypes, current use of stimulant medication, and comorbid ODD and phobia did not differ in boys and girls with ADHD. Demographic information is summarized in Table 1.

### 3.2 Prefrontal cortex analysis

#### 3.2.1 Cortical thickness (CT)

A 2 Diagnosis × 2 Sex × 2 Hemisphere × 5 ROI repeated measures ANOVA for CT of the PFC ROIs (ACC, DLPFC, ILPFC, mPFC, and OFC) revealed no significant effect of diagnosis, *F*(1,222) < 1, *p* = .947, or Diagnosis × Sex interaction, *F*(1,222) = 0.24, *p* = .625. There was a significant Diagnosis × ROI interaction, *F*(4,219) = 2.9, *p* = .023, ηp^2^ = .05, that was qualified by a significant Diagnosis × Hemisphere × ROI interaction, *F*(4,219) = 2.8, *p* = .026, ηp^2^ = .049. Examination of post-hoc tests comparing diagnostic groups for each ROI within each hemisphere revealed no significant differences between ADHD and TD groups for any of the ROIs, *p*-values > .30, although there was a trend for reduced ACC CT in the ADHD group, *p* = .070, which may have driven this interaction.

#### 3.2.2 Surface area (SA)

**Fig. 1:**
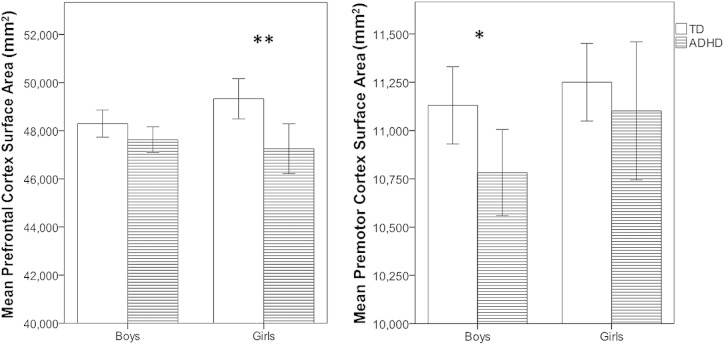
Left, reductions in PFC surface area were observed in girls with ADHD, compared to TD girls, but not boys. Right, reductions in PMC SA observed in boys with ADHD, compared to TD boys, but not girls. The error bars represent 95% confidence intervals and a single asterisk represents *p* < 0.05 and a double asterisk represents *p* < 0.01.

A 2 Diagnosis × 2 Sex × 2 Hemisphere × 5 ROI repeated measures ANOVA for SA of PFC ROIs (ACC, DLPFC, ILPFC, mPFC, and OFC) revealed a significant effect of diagnosis (TD > ADHD), *F*(1,222) = 13.6, *p* < .001, ηp^2^ = .058. This effect of diagnosis differed for boys and girls as evidenced by a marginal Diagnosis × Sex interaction, *F*(1,222) = 3.59, *p* = .059, ηp^2^ = .016, such that the effect of diagnosis was significant for girls, *F*(1,222) = 11.4, *p* = .001, ηp^2^ = .049, but not for boys, *F*(1,222) = 2.57, *p* = .110, ηp^2^ = .011 (see Fig. 1).

These effects were qualified by a significant Diagnosis × Sex × Hemisphere × ROI interaction, *F*(4,219) = 2.8, *p* = .026, ηp^2^ = .049. Post-hoc tests revealed that girls with ADHD showed smaller cortical SA in a much wider distribution of PFC regions than did boys with ADHD, including bilaterally in the DLPFC, left DLPFC: *F*(1,222) = 6.4, *p* = .012, ηp^2^ = .028; right DLPFC: *F*(1,222) = 5.0, *p* = .026, ηp^2^ = .022, as well as left ILPFC, *F*(1,222) = 6.4, *p* = .012, ηp^2^ = .028, left ACC, *F*(1,222) = 8.1, *p* = .005, ηp^2^ = .035, right mPFC, *F*(1,222) = 7.8, *p* = .006, ηp^2^ = .034, and right OFC, *F*(1,222) = 5.21, *p* = .023, ηp^2^ = .023. In contrast, boys with ADHD showed fewer PFC reductions compared to TD boys than did girls with ADHD, including the left mPFC, *F*(1,222) = 3.9, *p* = .050, ηp^2^ = .017, right ACC, *F*(1,222) = 9.4, *p* = .002, ηp^2^ = .041, and right OFC, *F*(1,222) = 3.7, *p* = .055, ηp^2^ = .016. Pairwise comparisons are summarized in Table 2.

### 3.3 Premotor cortex analysis

#### 3.3.1 Cortical thickness

A 2 Diagnosis × 2 Sex × 2 Hemisphere × 3 ROI repeated measures ANOVA for the PMC ROIs (SMC, LPM, FEF) revealed no significant main effect of Diagnosis, *F*(1,222) < 1, *p* = .679, and no Diagnosis × Sex interaction, *F*(1,222) < 1, *p* = .807. Additionally, all other interactions with Diagnosis were not significant.

#### 3.3.2 Surface area

A 2 Diagnosis × 2 Sex × 2 Hemisphere × 3 ROI repeated measures ANOVA for the PMC ROIs (SMC, LPM, FEF) revealed a marginally significant main effect of diagnosis (TD > ADHD), *F*(1,222) = 3.7, *p* = .057, ηp^2^ = .016. The effect of diagnosis did not significantly vary by sex, Diagnosis × Sex interaction: *F*(1,222) = 0.6, *p* = .441, although it was qualified by a Diagnosis × ROI interaction, *F*(2,221) = 5.2, *p* = .006, ηp^2^ = .045. Post-hoc tests revealed that SA was reduced in children with ADHD in the LPM, *F*(1,222) = 4.2, *p* = .042, ηp^2^ = .018, and SMC, *F*(1,222) = 3.9, *p* = .05, ηp^2^ = .017.

Given the pattern of findings for the PFC and our interest in sex differences in ADHD boys and girls compared to their sex-matched peers, separate analyses (Diagnosis × Hemisphere × ROI) for each sex were also conducted to determine whether the Diagnosis × ROI interaction reported above for surface area was present among boys and girls (2 additional models). These analyses revealed a significant effect of diagnosis for boys, *F*(1,153) = 5.2, *p* = .024, ηp^2^ = .033, but not for girls, *F*(1,69) = 0.6, *p* = .433, ηp^2^ = .009, suggesting that PMC SA is reduced in boys with ADHD compared to TD boys, but not in girls with ADHD compared to TD girls (see Fig. 1). For boys, there was a significant Diagnosis × ROI interaction, *F*(2,152) = 3.6, *p* = .03, ηp^2^ = .045, such that boys with ADHD showed significantly smaller cortical SA only in the bilateral LPM, *F*(1,153) = 8.4, *p* = .004, ηp^2^ = .052. For girls, there was also a Diagnosis × ROI interaction, *F*(2,68) = 4.9, *p* = .011, ηp^2^ = .13, such that girls with ADHD showed smaller cortical SA only in the bilateral SMC only, *F*(1,69) = 4.1, *p* = .046, ηp^2^ = .056. Pairwise comparisons are summarized in Table 2.

### 3.4 Effects of ODD and medication status

For the PFC, separate analyses of children with ADHD with (n=35) and without (n=58) comorbid ODD compared to TD children revealed that for the ADHD without ODD group there was an effect of ADHD Diagnosis, *F*(1,186) = 7.9, *p* = .005, ηp^2^ = .04, as well as a significant Diagnosis × Sex × Hemisphere × ROI interaction, *F*(4, 183) = 3.2, *p* = .014, ηp^2^ = .07. In contrast, for children with comorbid ODD, while there was a main effect of Diagnosis on PFC SA, *F*(1, 165) = 8.7, *p* = .004, ηp^2^ = .05, there was no Diagnosis × Sex × Hemisphere × ROI interaction, *F*(4, 162) = 1.2, *p* = .297, ηp^2^ = .03. These results suggest that the diagnosis of ADHD, and not the presence of comorbid ODD, is driving the interaction findings observed in the whole group. There was also an effect of medication status for the PFC such that among the children currently prescribed with stimulant medication (n=65), a significant effect of diagnosis, *F*(1,195) = 13.9, *p* < .001, ηp^2^ = .07, and a marginal Diagnosis × Sex interaction, *F*(1,195) = 3.6, *p* = .058, ηp^2^ = .02, was observed, similar to the findings for the whole group. In contrast, these effects were not present among those that were not currently prescribed stimulant medication (n=28), diagnosis: *F*(1,156) = 2.6, *p* = .108, ηp^2^ = .02, Diagnosis × Sex: *F*(1,156) = .92, *p* = .340, ηp^2^ < .01. Analysis of ODD and medication status on PFC cortical thickness revealed no effect of ODD or medication status.

Similarly, for the PMC, separate analyses of children with ADHD with and without comorbid ODD compared to TD children revealed that for the ADHD without ODD group, there was an effect of ADHD diagnosis, *F*(1,186) = 7.7, *p* = .006, ηp^2^ = .04, as well as a significant Diagnosis × ROI interaction, *F*(2,185) = 5.0, *p* = .008, ηp^2^ = .05. In contrast, for children with ODD, there was no significant effect of diagnosis, *F*(1,165) = .05, *p* = .83, ηp^2^ < .001, or a Diagnosis × ROI interaction, *F*(2,164) = 2.4, *p* = .091, ηp^2^ = .03. These results suggest that the diagnosis of ADHD, and not the presence of comorbid ODD, is driving the findings observed in the whole group. Analyses of the effect of stimulant medication status on the PM SA revealed that both groups showed a significant Diagnosis × ROI interaction (currently not prescribed medication: *F*[2,155] = 4.0, *p* = .021, ηp^2^ = .05; currently prescribed medication: *F*[2,194] = 3.3, *p* = .038, ηp^2^ = .03), suggesting that medication status had no impact on PMC SA findings from the whole group. Additionally, there were no significant main effects or interactions observed in any of the ODD or medication status subgroups for PMC cortical thickness.

### 3.5 Brain behavior correlation

Partial correlations were used to assess relationships between Ranta atlas ROIs and symptom severity (CPRS-R:L ADHD Total score) in children with ADHD. Although age was only correlated with PMC SA (bilateral PMC SA: *r* = .232, *p* = .026) but not PFC SA (bilateral PFC SA: *r* = .162, *p* = .121), age was included as a covariate in both PMC and PFC associations with symptom severity to be consistent.

**Fig. 2:**
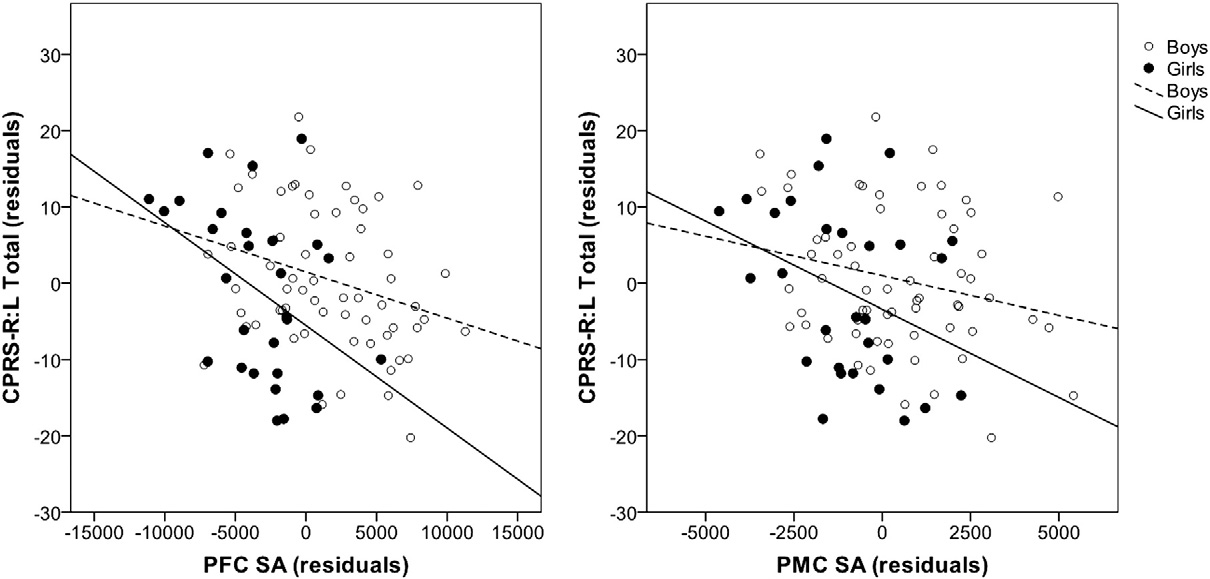
The scatter plots illustrate the inverse relationship between PFC (left) and PMC (right) SAs and Conners' Parent Rating Scale-Revised Long Version Total Score while covarying for age (partial correlation). PFC was significantly associated with symptom severity in both girls and boys (boys: *r* = –.289, *p* = .023; *r* = –.441, *p* = .019). PMC SA was marginally associated with symptom severity in boys (*r* = –.244, *p* = .056), whereas it was not significant in girls (*r* = –.266, *p* = .171). Filled circles and solid lines represent girls while unfilled circles and dotted lines represent boys.

In the overall sample of children with ADHD we found that greater total PFC and PMC SA was significantly associated with lower ADHD symptom severity (PFC: *r* = –.252, *p* = .016; PMC: *r* = –.219, *p* = .037). Follow-up analyses exclusively investigated symptom correlations with SA in each of the ROIs for each sex. Among boys, PMC SA was associated with symptom severity (*r* = –.244, *p* = .056), whereas this correlation was not significant for girls with ADHD (*r* = –.266, *p* = .171). However, it should be noted that the correlation coefficient was similar for boys and girls with ADHD, suggesting that the lack of significance among girls with ADHD may be due to reduced power. In contrast, PFC SA was significantly associated with symptom severity in both girls and boys with ADHD (girls: *r* = –.441, *p* = .019; boys: *r* = –.289, *p* = .023), although this relationship appears to be stronger among girls with ADHD (Fig. 2). Correlation statistics are summarized in Table 3.

## 4 Discussion

The primary focus of this study was to examine neuroanatomical differences in the frontal lobe in 8–12 year-old boys and girls with ADHD in comparison to same-sex TD children. To accomplish this, we included a large sample of both TD and ADHD boys and girls and employed an automated parcellation of functionally distinct subdivisions of the frontal lobe (Ranta et al., 2014). In line with previous research, we found reductions in frontal lobe SA in school-age (pre-adolescent) children with ADHD, whereas no significant differences in CT were observed. Interestingly, differential patterns of reduced SA relative to TD children were observed for girls with ADHD compared to boys with ADHD. Boys with ADHD, compared to TD boys, showed posterior (premotor cortex; PMC) decreases in SA, whereas girls with ADHD showed more anterior (prefrontal cortex; PFC) decreases in SA when compared to TD girls. These findings should be considered in light of the different neurodevelopmental trajectories for both diagnostic groups and sexes, such that girls develop earlier than boys (Lenroot et al., 2007) and TD children develop earlier than ADHD children (Shaw et al., 2007). Therefore, higher order association areas that develop last, such as anterior regions of the frontal lobe, may show differences in the girls but not boys because boys, both TD and ADHD, have not undergone this advanced stage of cortical development. Additionally, although approximately 40% of our sample had comorbid ODD, follow-up analysis revealed that the results presented at the whole group level for both PFC and PMC were principally driven by ADHD, and not ODD, status. Furthermore, approximately 70% of the ADHD sample were prescribed stimulant medication at the time of the study. Follow-up analyses indicated that there was no significant effect of medication status on PMC SA, yet PFC SA differences were more pronounced in those currently prescribed stimulants. This may suggest more atypical prefrontal morphometry or greater power to detect these differences among the sample of children currently prescribed stimulant medication compared to those that are not, relative to TD children. Although our sample was relatively large, it was still not sufficient to examine the impact of medication on Diagnosis × Sex interactions (only 10 ADHD females were not currently prescribed stimulant medication). Therefore our findings regarding the impact of stimulant medication on the effect of sex in ADHD should be considered preliminary.

Reductions in PMC SA in boys with ADHD are in line with previous research showing impairments in motor function (Cole et al., 2008; MacNeil et al., 2011; Mostofsky et al., 2003), basic motor response inhibition and preparation (O'Brien et al., 2010), and reductions in PMC volume (Mostofsky et al., 2002). These anatomical differences may be relevant to ADHD-related sex differences in motor impairments, such that boys with ADHD in this age range (8–12 years old) show a greater propensity to produce mirror overflow than do girls with ADHD, who do not show increased mirror overflow compared to TD girls (MacNeil et al., 2011). Mirror overflow movements in boys with ADHD, thought to reflect a maturational delay of intracortical inhibition, are in line with research by Shaw and colleagues showing a maturational delay in cortical development in ADHD (Shaw et al., 2007).

Girls with ADHD showed more distributed decreases in SA in the PFC compared to ADHD boys. These results are in line with neuropsychological testing showing that girls with ADHD present with impairments in planning/switching (O'Brien et al., 2010) but do not show significant impairments in motor control relative to age matched TD girls (Cole et al., 2008; MacNeil et al., 2011). Our neuroanatomical findings suggest that higher order deficits in planning and greater propensity for internalizing behaviors in girls with ADHD (Lahey et al., 2007) may be related to decreased SA in the mPFC and DLPFC (planning and switching) and OFC (emotion regulation). The greater distribution of significant reductions in the PFC in girls with ADHD may reflect a general delay in cortical development (compared to TD girls) but, like all girls, a relative advancement of cortical development compared to ADHD boys.

Although boys and girls with ADHD showed different patterns of reductions in SA, regions such as the OFC, mPFC, and ACC were reduced in both boys and girls with ADHD. These regions, given their connections with the ventral striatum (Haber and Knutson, 2010), are part of the brain reward circuitry and are involved in integrating cognitive and motivational processes. Etiological theories of ADHD have emphasized the interaction of cognition and motivation (Castellanos et al., 2006) as underlying the behavioral dysregulation that characterizes this disorder. Specifically, children with ADHD tend to display atypical reward-based decision-making (i.e., delay discounting; e.g., Sonuga-Barke and Fairchild, 2012), motivation/response to reinforcement (e.g., Luman et al., 2010), and emotion regulation (Shaw et al., 2014). Studies have shown that the OFC is involved in the inhibition of inappropriate emotional responses (Itami and Uno, 2002) and identifying the reinforcing value of stimuli (Chib et al., 2009). The ACC is also thought to be an interface between cognition and motivation and is involved in reward processing and error or conflict awareness (Holroyd and Yeung, 2011). Research has also shown that the mPFC is associated with reward processing, executive control, error detection, conflict monitoring, and reward-guided learning (Euston et al., 2012). Furthermore, resting-state fMRI has implicated the mPFC as a central node in the default mode network (DMN), which has been suggested to be a network that emerges during non-task related, self-reflective periods. Previous work from Castellanos and colleagues has shown a decreased coherence in the DMN and that a decreased DMN suppression is associated with intra-individual variability (Uddin et al., 2008). Thus, our findings of reduced mPFC SA in boys and girls with ADHD might suggest an anatomical basis for an immature DMN, in support of the idea of a developmental delay in ADHD (Fair et al., 2010).

In line with diagnostic group differences in SA, increased SA (more typical SA) was associated with decreased ADHD symptom severity within the ADHD group. Additionally, PMC and PFC SAs were associated with ADHD symptom severity among boys, whereas PMC SA was not significantly associated with symptom severity among girls with ADHD, although the correlation coefficient was similar to that of boys suggesting a lack of power, and a strong association between PFC SA and symptom severity, was observed. Similar to the PFC SA diagnostic group differences seen in boys and girls with ADHD, a stronger association between PFC SA and symptom severity may be related to the earlier development of the PMC relative to the PFC and of girls relative to boys, such that ADHD-associated differences in PFC SA seen in the girls may predate that of boys. Previous work by Shaw and colleagues has shown that the rate of cortical thinning is associated with symptom severity as well as syndromatic and symptomatic remission (Shaw et al., 2011). In this study we find that a decrease in SA is associated with greater symptom severity while controlling for age. It is possible that children in our sample who show decreased SA may show a greater perturbation in their cortical development and therefore present with greater symptom severity, an interpretation that is consistent with research by Shaw (Shaw et al., 2006). Furthermore, a sex difference in the developmental trajectory of cortical folding is a likely mechanism underlying this association, but longitudinal studies would be needed in order to understand whether boys with ADHD show similar differences in PFC SA as do girls with ADHD at a later point in development.

To our knowledge, only one other study has investigated sex differences in frontal lobe morphology in children with ADHD (Mahone et al., 2011). Although both studies found similar frontal lobe results, decreases in ADHD children compared to TD children, some differences were observed at the ROI level, as the prior study found reduced SMC volume in both girls and boys with ADHD with sexual dimorphic findings only for lateral PMC (only girls) and medial PFC (only boys). These differences may be the result of using different parcellation methods as well as the use of different cortical metrics. Mahone et al. used manually delineated ROIs to extract gray and white matter volume. In the current study, an automated parcellation, based on the aforementioned manual parcellation, was used to extract SA and CT, not volume. Additionally, this study had larger group sizes (64 ADHD boys compared to 21, and 29 ADHD girls compared to 21) that may also contribute to the differences in our results. In particular, the larger group sizes and focus on more specific metrics may have been useful in detecting a wider distribution of PFC abnormalities in girls with ADHD.

Due to the limited research on sex differences in children with ADHD, we chose to focus our investigation on PMC and PFC regions in a group of relatively “pure ADHD” children (i.e., without significant comorbidities). The ROI based approach used in this study allowed us to investigate functional subdivision of the frontal lobe, which has not been done. By doing so, we were unable to localize the clusters within the ROI that drove these sex and diagnostic differences. Additionally, the inclusion of hemisphere in our analysis revealed significant differences between boys and girls with ADHD, yet no clear laterality effect was observed. Although our data suggest differences in frontal lobe cortical development in boys and girls with ADHD compared to their TD peers, we were unable to test those differences in cortical development due to our cross-sectional data. Previous longitudinal research has shown a general reduction in volume across all lobes as well cerebellum in ADHD subjects ages 5–19 (Castellanos et al., 2002). Furthermore, the use of a “pure ADHD” sample may reduce our ability to generalize our results. Larger group sizes will allow for further investigation into the effect of comorbidities in ADHD as well as the effect of stimulant medication. However, our findings provide an important foundation for future research, which can expand upon the methods and sample characteristics for the current study.

This study emphasizes the need for the investigation of sex-differences in ADHD, particularly in terms of brain structure and function, cognition, and behavior. In this study we were able to identify sex-based differences in SA, such that boys with ADHD showed more prominent PMC reductions in SA, while girls with ADHD showed more prominent PFC reductions in SA. We also found that greater SA was associated with lower ADHD symptom severity. Future research is needed to clarify the following: 1) whether boys and girls with ADHD follow the same developmental trajectory in SA, but with girls somewhat ahead of boys, and 2) how these reductions in SA are related to changing developmental cognitive and behavioral impairments. A greater understanding of sex differences in ADHD and trajectories of brain development and functional outcomes has important implications for possible remediation or therapies tailored differentially to boys and girls with ADHD.

## Figures and Tables

**Table 1:** Demographic statistics.

	TD						ADHD						ADHD vs. TD *p*-values		
	Girls (N = 42)		Boys (N = 91)		All (N = 133)		Girls (N = 29)		Boys (N = 64)		All (N = 93)		Girls	Boys	All
	Mean	SD	Mean	SD	Mean	SD	Mean	SD	Mean	SD	Mean	SD			
Age (years)	10.0	0.94	10.4	1.3	10.3	1.2	10.3	1.6	10.1	1.4	10.2	1.4	.433	.278	.606
% minority	31%		26%		28%		24%		34%		31%		.283	.530	.584
SES	52	10	52	10	52	10	52	10	50	11	51	11	.895	.303	.346
Handedness	0.79	0.33	0.63	0.58	0.68	0.52	0.73	0.42	0.65	0.55	0.68	0.51	.476	.859	.923
FSIQ	111	10	115	13	114	12	108	12	107	12	106	13	.049	<.001	<.001
VCI	113	12	121	14	118	14	111	13	113	15	111	15	.096	.001	<.001
PRI	106	10	112	14	110	13	105	13	109	13	108	13	.806	.193	.230
WMI	105	11	109	14	108	13	104	13	101	13	101	11	.375	.001	.001
PSI	105	15	100	13	102	14	102	15	91	10	93	11	.007	<.001	<.001
													ADHD boys vs. ADHD girls *p*-values		
CPRS:R–L total raw	4	3	6	5	5	5	32	11	33	9	33	10			.427
ADHD subtype, CO:IA:HI (count)	0		0		0		20:8:1		49:13:2		69:21:3				.730
% stimulant medication							69%		70%		70%				.896
ODD (count)	0		0		0		13		22		35				.335
Phobia (count)	3		2		5		5		7		12				.401

Note. % minority = percentage of subjects with a self-reported race of African American, Asian, Hispanic, or biracial; SES = Hollingshead Four-Factor Index of Socioeconomic Status; handedness = Edinburgh Handedness Inventory; FSIQ = Wechsler Intelligence Scale for Children Fourth Edition (WISC-IV) Full-scale IQ, VCI = WISC-IV verbal comprehension index, PRI = WISC-IV perceptual reasoning index, WMI = WISC-IV working memory index, PSI = WISC-IV processing speed index; CPRS:R–L = Conners' Parent and Teacher Rating Scales-Revised Long Version; CO = combined subtype; IA = inattentive subtype; HI = Hyperactive/impulsive subtype; % stimulant medication = percentage of subjects taking stimulant medication at the time of the study (all subjects discontinued medication the day prior to and day of study participation); ODD = Oppositional Defiant Disorder.

**Table 2:** Total brain volume and frontal lobe cortical surface area.

	TD						ADHD						ADHD vs. TD differences	
	Girls		Boys		All		Girls		Boys		All			
	Mean	SD	Mean	SD	Mean	SD	Mean	SD	Mean	SD	Mean	SD	Girls	Boys
Total brain volume (mm^3^)	990,656	82,200	1,096,920	100,946	1,063,363	107,261	981,137	86,723	1,074,734	90,312	1,045,548	98,866		
Surface area (mm^2^) Prefrontal ROIs														
ACC	5148	521	4909	428	4984	471	4885	435	4752	471	4794	461	(L)**	(R)**
DLPFC	14,389	1248	14,053	1224	14,159	1237	13,600	1291	14,116	1077	13,955	1165	(B)*	
ILPFC	7611	825	7530	875	7555	857	7234	687	7393	752	7344	733	(L)*	
mPFC	9736	614	9559	752	9615	714	9305	867	9385	683	9360	742	(R)**	(L)*
OFC	12,444	799	12,246	952	12,309	909	12,227	872	11,983	860	12,059	866	(R)*	
Premotor ROIs														
FEF	1885	181	1871	292	1876	261	1969	241	1856	281	1882	266		
LPM	5627	430	5634	605	5632	554	5570	603	5350	597	5544	583		(B_w_)**
SMC	3738	357	3625	441	3661	418	3563	356	3576	346	3624	392	(B_w_)*	

ACC = Anterior cingulate, DLPFC = dorsolateral prefrontal cortex, ILPFC = inferior lateral prefrontal cortex, mPFC = medial prefrontal cortex, OFC = orbitofrontal cortex; FEF = frontal eye field, LPM = lateral premotor cortex, SMC = supplementary motor cortex; +*p* ≤ 0.1; L = left, R = right, B = left and right, B_w_ = bilateral ROI was significant and laterality effects were not tested.

* *p* ≤ 0.05.

** *p* ≤ 0.01.

**Table 3:** Partial correlation coefficients for frontal lobe ROI morphology and symptom severity.

		Surface area						Surface area			
		Total prefrontal	ACC	DLPFC	ILPFC	mPFC	OFC	Total premotor	FEF	LPM	SMC
Girls	CPRS:R	−.441*	−.359+	−.369+	−.407*	−.379*	−.223	−.266	−.108	−.303	−.173
Boys	CPRS:R	−.289*	−.192	−.176	−.229+	−.241+	−.312*	−.244+	−.167**	−.168	−.293*

Note: Partial correlation coefficients were calculated in the ADHD group only while covarying for age. CPRS: R–L = Conners' Parent Rating Scale-Revised Long Version ADHD Total Score; ACC = anterior cingulate, DLPFC = dorsolateral prefrontal cortex, ILPFC = inferior lateral prefrontal cortex, mPFC = medial prefrontal cortex, OFC = orbitofrontal cortex; FEF = frontal eye field, LPM = lateral premotor cortex, SMC = supplementary motor cortex; +*p* ≤ 0.1, *p ≤ 0.05, and **p ≤ 0.01.

## References

[bb001] Almeida Montes L.G., Prado Alcántara H., Martínez García R.B., De La Torre L.B., Avila Acosta D., Duarte M.G. (2013). Brain cortical thickness in ADHD: age, sex, and clinical correlations. J. Atten. Disord..

[bb002] Arnsten A.F. (2009). Toward a new understanding of attention-deficit hyperactivity disorder pathophysiology: an important role for prefrontal cortex dysfunction. CNS Drugs.

[bb003] Berquin P.C., Giedd J.N., Jacobsen L.K., Hamburger S.D., Krain A.L., Rapoport J.L., Castellanos F.X. (1998). Cerebellum in attention-deficit hyperactivity disorder: a morphometric MRI study. Neurol..

[bb004] Castellanos F.X., Giedd J.N., Berquin P.C., Walter J.M., Sharp W., Tran T., Vaituzis A.C., Blumenthal J.D., Nelson J., Bastain T.M., Zijdenbos A., Evans A.C., Rapoport J.L. (2001). Quantitative brain magnetic resonance imaging in girls with attention-deficit/hyperactivity disorder. Arch. Gen. Psychiatry.

[bb005] Castellanos F.X., Lee P.P., Sharp W., Jeffries N.O., Greenstein D.K., Clasen L.S., Blumenthal J.D., James R.S., Ebens C.L., Walter J.M., Zijdenbos A., Evans A.C., Giedd J.N., Rapoport J.L. (2002). Developmental trajectories of brain volume abnormalities in children and adolescents with attention-deficit/hyperactivity disorder. JAMA.

[bb006] Castellanos F.X., Sonuga-Barke E.J., Milham M.P., Tannock R. (2006). Characterizing cognition in ADHD: beyond executive dysfunction. Trends Cogn. Sci..

[bb007] Chib V.S., Rangel A., Shimojo S., O'Doherty J.P. (2009). Evidence for a common representation of decision values for dissimilar goods in human ventromedial prefrontal cortex. J. Neurosci..

[bb008] Cole W.R., Mostofsky S.H., Larson J.C., Denckla M.B., Mahone E.M. (2008). Age-related changes in motor subtle signs among girls and boys with ADHD. Neurology.

[bb009] Conners C.K., Sitarenios G., Parker J.D., Epstein J.N. (1998). The revised Conners' Parent Rating Scale (CPRS-R): factor structure, reliability, and criterion validity. J. Abnorm. Child Psychol..

[bb0010] Denckla M.B., Rudel R.G. (1978). Anomalies of motor development in hyperactive boys. Ann. Neurol..

[bb0011] DuPaul G., Power T., Anastopoulos A., Reid R. (1998). ADHD Rating Scal-IV.

[bb0012] Euston D.R., Gruber A.J., McNaughton B.L. (2012). The role of medial prefrontal cortex in memory and decision making. Neuron.

[bb0013] Ewen J.B., Moher J.S., Lakshmanan B.M., Ryan M., Xavier P., Crone N.E., Denckla M.B., Egeth H., Mahone E.M. (2012). Multiple task interference is greater in children with ADHD. Dev. Neuropsychol..

[bb0014] Fair D.A., Posner J., Nagel B.J., Bathula D., Dias T.G., Mills K.L., Blythe M.S., Giwa A., Schmitt C.F., Nigg J.T. (2010). Atypical default network connectivity in youth with attention-deficit/hyperactivity disorder. Biol. Psychiatry.

[bb0015] Fischl B., van der Kouwe A., Destrieux C., Halgren E., Ségonne F., Salat D.H., Busa E., Seidman L.J., Goldstein J., Kennedy D., Caviness V., Makris N., Rosen B., Dale A.M. (2004). Automatically parcellating the human cerebral cortex. Cereb. Cortex.

[bb0016] Goldberg E., Roediger D., Kucukboyaci N.E., Carlson C., Devinsky O., Kuzniecky R., Halgren E., Thesen T. (2013). Hemispheric asymmetries of cortical volume in the human brain. Cortex J. Devoted Study Nerv. Syst. Behav..

[bb0017] Haber S.N., Knutson B. (2010). The reward circuit: linking primate anatomy and human imaging. Neuropsychopharmacol. Off. Publ. Am. Coll. Neuropsychopharmacol..

[bb0018] Hinshaw S.P., Carte E.T., Fan C., Jassy J.S., Owens E.B. (2007). Neuropsychological functioning of girls with attention-deficit/hyperactivity disorder followed prospectively into adolescence: evidence for continuing deficits?. Neuropsychol..

[bb0019] Hinshaw S.P., Owens E.B., Sami N., Fargeon S. (2006). Prospective follow-up of girls with attention-deficit/hyperactivity disorder into adolescence: evidence for continuing cross-domain impairment. J. Consult. Clin. Psychol..

[bb0020] Holroyd C., Yeung N. (2011). An integrative theory of anterior cingulate cortex function: option selection in hierarchical reinforcement learning. Neural Basis of Motivational and Cognitive Control.

[bb0021] Itami S., Uno H. (2002). Orbitofrontal cortex dysfunction in attention-deficit hyperactivity disorder revealed by reversal and extinction tasks. Neuroreport.

[bb0022] Jensen P.S., Rubio-Stipec M., Canino G., Bird H.R., Dulcan M.K., Schwab-Stone M.E., Lahey B.B. (1999). Parent and child contributions to diagnosis of mental disorder: are both informants always necessary?. J. Am. Acad. Child Adolesc. Psychiatry.

[bb0023] Koelkebeck K., Miyata J., Kubota M., Kohl W., Son S., Fukuyama H., Sawamoto N., Takahashi H., Murai T. (2014). The contribution of cortical thickness and surface area to gray matter asymmetries in the healthy human brain. Hum. Brain Mapp..

[bb0024] Kramer J.H., Quitania L., Dean D., Neuhaus J., Rosen H.J., Halabi C., Weiner M.W., Magnotta V.A., Delis D.C., Miller B.L. (2007). Magnetic resonance imaging correlates of set shifting. J. Int. Neuropsychol. Soc. JINS.

[bb0025] Lahey B.B., Hartung C.M., Loney J., Pelham W.E., Chronis A.M., Lee S.S. (2007). Are there sex differences in the predictive validity of DSM-IV ADHD among younger children?. J. Clin. Child Adolesc. Psychol. Off. J. Soc. Clin. Child Adolesc. Psychol. Am. Psychol. Assoc. Div. 53.

[bb0026] Lenroot R.K., Gogtay N., Greenstein D.K., Wells E.M., Wallace G.L., Clasen L.S., Blumenthal J.D., Lerch J., Zijdenbos A.P., Evans A.C., Thompson P.M., Giedd J.N. (2007). Sexual dimorphism of brain developmental trajectories during childhood and adolescence. Neuroimage.

[bb0027] Luman M., Tripp G., Scheres A. (2010). Identifying the neurobiology of altered reinforcement sensitivity in ADHD: a review and research agenda. Neurosci. Biobehav. Rev..

[bb0028] MacNeil L.K., Xavier P., Garvey M.A., Gilbert D.L., Ranta M.E., Denckla M.B., Mostofsky S.H. (2011). Quantifying excessive mirror overflow in children with attention-deficit/hyperactivity disorder. Neurology.

[bb0029] Mahone E.M. (2012). Neuropsychiatric differences between boys and girls with ADHD. Psychiatr. Times.

[bb0030] Mahone E.M., Ranta M.E., Crocetti D., O'Brien J., Kaufmann W.E., Denckla M.B., Mostofsky S.H. (2011). Comprehensive examination of frontal regions in boys and girls with attention-deficit/hyperactivity disorder. J. Int. Neuropsychol. Soc..

[bb0031] Montes L.G., Ricardo-Garcell J., De la Torre L.B., Alcántara H.P., García R.B., Acosta D.A., Bouzas A.F. (2011). Cerebellar gray matter density in females with ADHD combined type: a cross-sectional voxel-based morphometry study. J. Atten. Disord..

[bb0032] Mostofsky S.H., Cooper K.L., Kates W.R., Denckla M.B., Kaufmann W.E. (2002). Smaller prefrontal and premotor volumes in boys with attention-deficit/hyperactivity disorder. Biol. Psychiatry.

[bb0033] Mostofsky S.H., Newschaffer C.J., Denckla M.B. (2003). Overflow movements predict impaired response inhibition in children with ADHD. Percept. Mot. Skills.

[bb0034] Mostofsky S.H., Reiss A.L., Lockhart P., Denckla M.B. (1998). Evaluation of cerebellar size in attention-deficit hyperactivity disorder. J. Child Neurol..

[bb0035] O'Brien J.W., Dowell L.R., Mostofsky S.H., Denckla M.B., Mahone E.M. (2010). Neuropsychological profile of executive function in girls with attention-deficit/hyperactivity disorder. Arch. Clin. Neuropsychol..

[bb0036] Qiu A., Crocetti D., Adler M., Mahone E.M., Denckla M.B., Miller M.I., Mostofsky S.H. (2009). Basal ganglia volume and shape in children with attention deficit hyperactivity disorder. Am. J. Psychiatry.

[bb0037] Ranta M.E., Chen M., Crocetti D., Prince J.L., Subramaniam K., Fischl B., Kaufmann W.E., Mostofsky S.H. (2014). Automated MRI parcellation of the frontal lobe. Hum. Brain Mapp..

[bb0038] Ranta M.E., Crocetti D., Clauss J.A., Kraut M.A., Mostofsky S.H., Kaufmann W.E. (2009). Manual MRI parcellation of the frontal lobe. Psychiatry Res.

[bb0040] Reich W., Welner Z., Herjanic B. (1997). Diagnostic Interview for Children and Adolescents-IV.

[bb0041] Rubia K., Taylor E., Smith A.B., Oksanen H., Overmeyer S., Newman S., Oksannen H. (2001). Neuropsychological analyses of impulsiveness in childhood hyperactivity. Br J Psychiatry.

[bb0042] Rucklidge J.J. (2010). Gender differences in attention-deficit/hyperactivity disorder. Psychiatr. Clin. North Am..

[bb0043] Seidman L.J., Valera E.M., Makris N. (2005). Structural brain imaging of attention-deficit/hyperactivity disorder. Biol. Psychiatry.

[bb0044] Shaw P., Eckstrand K., Sharp W., Blumenthal J., Lerch J.P., Greenstein D., Clasen L., Evans A., Giedd J., Rapoport J.L. (2007). Attention-deficit/hyperactivity disorder is characterized by a delay in cortical maturation. Proc. Natl. Acad. Sci. U. S. A..

[bb0045] Shaw P., Gilliam M., Liverpool M., Weddle C., Malek M., Sharp W., Greenstein D., Evans A., Rapoport J., Giedd J. (2011). Cortical development in typically developing children with symptoms of hyperactivity and impulsivity: support for a dimensional view of attention deficit hyperactivity disorder. Am. J. Psychiatry.

[bb0046] Shaw P., Lalonde F., Lepage C. (2009). Development of cortical asymmetry in typically developing children and its disruption in attention-deficit/hyperactivity disorder. Arch. Gen. Psychiatry.

[bb0047] Shaw P., Lerch J., Greenstein D., Sharp W., Clasen L., Evans A., Giedd J., Castellanos F.X., Rapoport J. (2006). Longitudinal mapping of cortical thickness and clinical outcome in children and adolescents with attention-deficit/hyperactivity disorder. Arch. Gen. Psychiatry.

[bb0048] Shaw P., Malek M., Watson B., Sharp W., Evans A., Greenstein D. (2012). Development of cortical surface area and gyrification in attention-deficit/hyperactivity disorder. Biol. Psychiatry.

[bb0049] Shaw P., Stringaris A., Nigg J., Leibenluft E. (2014). Emotion dysregulation in attention deficit hyperactivity disorder. Am. J. Psychiatry.

[bb0050] Sonuga-Barke E.J., Fairchild G. (2012). Neuroeconomics of attention-deficit/hyperactivity disorder: differential influences of medial, dorsal, and ventral prefrontal brain networks on suboptimal decision making?. Biol. Psychiatry.

[bb0051] Sowell E.R., Thompson P.M., Welcome S.E., Henkenius A.L., Toga A.W., Peterson B.S. (2003). Cortical abnormalities in children and adolescents with attention-deficit hyperactivity disorder. Lancet.

[bb0052] Uddin L.Q., Kelly A.M., Biswal B.B., Margulies D.S., Shehzad Z., Shaw D., Ghaffari M., Rotrosen J., Adler L.A., Castellanos F.X., Milham M.P. (2008). Network homogeneity reveals decreased integrity of default-mode network in ADHD. J. Neurosci. Methods.

[bb0053] Wechsler D. (2002). Wechsler Individual Achievement Test.

[bb0054] Wechsler, D., Wechsler Intelligence Scale for Children , fourth edition (2003). WISC-IV

[bb0055] Wodka E.L., Mostofsky S.H., Prahme C., Gidley Larson J.C., Loftis C., Denckla M.B., Mahone E.M. (2008). Process examination of executive function in ADHD: sex and subtype effects. Clin. Neuropsychol..

[bb0056] Wolosin S.M., Richardson M.E., Hennessey J.G., Denckla M.B., Mostofsky S.H. (2009). Abnormal cerebral cortex structure in children with ADHD. Hum. Brain Mapp..

